# Hierarchical effects on target detection and conflict monitoring

**DOI:** 10.1038/srep32234

**Published:** 2016-08-26

**Authors:** Bihua Cao, Feng Gao, Maofang Ren, Fuhong Li

**Affiliations:** 1 School of Psychology, JiangXi Normal University, NanChang 330022, P. R. China; 2 Research center of brain and cognitive neuroscience, Liaoning Normal University, Dalian 116029, P. R. China

## Abstract

Previous neuroimaging studies have demonstrated a hierarchical functional structure of the frontal cortices of the human brain, but the temporal course and the electrophysiological signature of the hierarchical representation remains unaddressed. In the present study, twenty-one volunteers were asked to perform a nested cue-target task, while their scalp potentials were recorded. The results showed that: (1) in comparison with the lower-level hierarchical targets, the higher-level targets elicited a larger N2 component (220–350 ms) at the frontal sites, and a smaller P3 component (350–500 ms) across the frontal and parietal sites; (2) conflict-related negativity (non-target minus target) was greater for the lower-level hierarchy than the higher-level, reflecting a more intensive process of conflict monitoring at the final step of target detection. These results imply that decision making, context updating, and conflict monitoring differ among different hierarchical levels of abstraction.

When we encounter multiple stimuli, we do not merely link together the elements of the string of stimuli as we encounter them^1^. Instead, we are likely to generate an abstract representation of the structure of the sequence, which describes how the stimuli are organized^2^. A hierarchical representation is a mental representation that consists of components and nested subcomponents, where the superordinate components exert an influence on actions represented at the subcomponent level^3^,^4^,^5^,^6^,^7^.

In recent years, growing evidence suggests that a functional organizing principle along the rostro-caudal axis of the frontal lobes is based on a hierarchy of action control^6^,^8^,^9^,^10^,^11^,^12^,^13^,^14^. The strongest evidence to date for a functional gradient along the rostro-caudal axis of the frontal lobes comes from neuroimaging studies in which the hierarchy of abstraction of response selection was progressively varied^9^,^12^. For example, Nee and Brown^13^ designed a nested series of contextual cues, of which higher hierarchy context cues denoted an abstract rule set while lower hierarchy context cues provided more concrete information^13^. The results showed that rostral areas of the lateral orbitofrontal cortex represented the higher hierarchy context, while the mid-dorsolateral prefrontal cortex and inferior frontal junction represented more concrete rules. The notion of a hierarchy is also supported by lesion studies and single-neuron recording studies^15^,^16^,^17^.

The purpose of the present study was to investigate the spatiotemporal patterns of brain activity associated with different hierarchical levels of target detection. The task was modified from Nee and Brown^13^. As shown in Fig. 1, at the higher level of the hierarchy some context cues (i.e., 1-***B*** and 2-***A***) clearly lead to a non-target response. By contrast, other context cues potentially lead to a target response (i.e., 1-***A*** and 2-***B***). At the lower level of the hierarchy, some context cues (i.e., 1-A-***X***) clearly lead to a target response, while others cue a non-target response. Therefore, the process of target detection is affected by two variables. One is the stimulus type (target vs. non-target) and the other is the hierarchy level (higher vs. lower).

First, we aimed to identify the ERP components that are correlated with hierarchical representation of context by comparing the ERPs elicited by the higher-level targets with those of the lower-level targets. Previous ERP studies suggest that a fronto-central negative ERP component, such as N2, is usually enhanced in conflict trials that demand an unexpected response^18^,^19^,^20^,^21^,^22^, and this conflict-related negativity (CRN) is typically evoked by non-targets in target detection tasks^23^,^24^,^25^, by negative feedback^26^,^27^,^28^, or by mismatched stimuli in perceptual template-matching tasks^29^,^30^,^31^,^32^,^33^,^34^. In the present study, conflict is defined as having multiple incompatible response pathways or a mismatch between the perceived stimulus and working memory or expectancy. We hypothesized that the conflict detected in matching a stimulus (e.g., B or Y) to the mental template (e.g., 1-A-X) might be greater at the lower hierarchical levels relative to the higher hierarchical levels, because the mental template has been consolidated before the stimuli were presented at the lower hierarchical level, or at the end of a sequence^29^. Consequently, CRNs elicited by non-targets are expected to be greater at lower hierarchical levels than higher hierarchical levels. For the targets, the conflict process is less intensive because the appearance of stimuli matches the expectation of participants well^7^,^13^. However, the hierarchy effect is also expected to be observed during the N2 time window, because more competing responses are cued by the higher-targets than by the lower-targets. According to the model of conflict monitoring proposed by Botvinick *et al*.^35^, the higher target presentation elicited a parallel activation of multiple incompatible response pathways, which are more likely to cause crosstalk during the period between stimulus presentation and response delivery. So, we hypothesized that higher targets might evoke a larger N2 than lower targets.

Second, P3 is often linked to the deployment of attentional resources and context updating in tasks that require stimulus evaluation and memory updating^36^,^37^,^38^. At the higher level, a target context cue (e.g., 1-***A***) cannot unambiguously assert the final target because the subsequent letter could be either X or Y. So, the cognitive context cannot be comprehensively updated. Compared with the higher hierarchical level, the context cues at lower hierarchical levels are more specific, and closer to the terminal sequences, such that memory can be more thoroughly updated. Therefore, we expected that the lower-level targets would elicit an increased P3 amplitude when compared with higher-level targets.

## Results

The response accuracies for target and non-target sequences were 96.2% and 97.1%, respectively. There was no significant main effect of stimulus type on the accuracies. After excluding incorrect trials and RTs that were greater than 3 standard deviations from the mean, the results indicated that the responses were significantly faster for the targets (347 ms) than for the non-targets (364 ms), *F* (1, 20) = 8.9, *p* < 0.01, η^2^ = 0.309.

ERPs were time-locked to letter onset and grand-averaged across all participants. As shown in Fig. 2, the N2 (220–350 ms) and P3 (350–500 ms) components were elicited by all four conditions (higher target, lower target, higher non-target, lower non-target).

The results of the ANOVA on the N2 latencies revealed a main effect of stimulus type, *F* (1, 20) = 17.0, *p* < 0.01, η^2^ = 0.486, with longer latency for the non-targets. There was a significant interaction between hierarchy and type, *F* (1, 20) = 24.0, *p* < 0.001, η^2^ = 0.572. Simple-effects tests indicated that the type effect was significant only at the lower-hierarchy level, *F* (1, 20) = 39.4, *p* < 0.001, η^2^ = 0.686, while the type effect was non-significant at the higher hierarchy level (*p* = 0.11).The hierarchy effect was only found for targets, *F* (1, 20) = 33.7, *p* < 0.001, η^2^ = 0.652 (Fig. 3). In comparison with the lower targets, the higher targets elicited a delayed N2 peak. For the non-targets, the hierarchical effect on the N2 latency was not found (*p* = 0.06).

For the N2 amplitudes, there was a main effect of type, with a larger N2 amplitude for non-targets as compared with targets, *F* (1, 20) = 16.2, *p* < 0.005, η^2^ = 0.474. An interaction between hierarchy and caudality was observed, *F* (2, 40) = 4.4, *p* < 0.03, η^2^ = 0.193. Simple-effects tests indicated that the hierarchy effect was observed only in the frontal regions, *F* (1, 20) = 5.3, *p* < 0.04, η^2^ = 0.227 (Fig. 4). There was an interaction between hierarchy and type, *F* (1, 20) = 10.5, *p* < 0.01, η^2^ = 0.369. Further analysis of the simple effects indicated that there was a hierarchy effect for the targets, *F* (1, 20) = 8.8, *p* < 0.01, η^2^ = 0.328; whereas the hierarchy effect was non-significant for non-targets (*p* = 0.09).

For P3 peak latencies, the ANOVA revealed a main effect of type, *F* (1, 20) = 56.0, *p* < 0.001, η^2^ = 0.757, with longer latencies for non-targets as compared with targets. There was an interaction between hierarchy and type, *F* (1, 20) = 9.6, *p* < 0.01, η^2^ = 0.349. Further analysis revealed that the type effect was markedly revealed at both the higher and lower hierarchy levels [*F*_lower_ (1, 20) = 106.0, *p* < 0.001, η^2^ = 0.855; *F*_higher_ (1, 20) = 12.6, *p* < 0.01, η^2^ = 0.411]. The hierarchy effect was only found for the non-targets, *F* (1, 20) = 6.9, *p* < 0.02, η^2^ = 0.278, with longer latency at the lower hierarchy; the hierarchy effect was non-significant for the targets (*p* = 0.19).

For the P3 amplitudes, there was a main effect of hierarchy, *F* (1, 20) = 121.6, *p* < 0.0001, η^2^ = 0.871, and a main effect of type, *F* (1, 20) = 4.6, *p* < 0.05, η^2^ = 0.202. There was an interaction between hierarchy and caudality, *F* (2, 40) = 15.9, *p* < 0.0001, η^2^ = 0.468. Simple effects tests indicated that the hierarchy effect distributed over all three regions [*F*_frontal_ (1, 20) = 81.5, *p* < 0.0001, η^2^ = 0.819; *F*_central_ (1, 20) = 136.3, *p* < 0.0001, η^2^ = 0.883; *F*_parietal_ (1, 20) = 58.9, *p* < 0.0001, η^2^ = 0.766]. There was an interaction between type and caudality, *F* (2, 40) = 9.4, *p* < 0.01, η^2^ = 0.344. Simple effects tests indicated that the type effect was found only in the frontal regions, *F* (1, 20) = 10.2, *p* < 0.01, η^2^ = 0.363. In the central and parietal regions, the type effects were non-significant (both *p*s > 0.05).

Regarding the hierarchy effect on the CRN, we followed the same method as Miltner *et al*.^39^. We subtracted the target waveform from the non-target waveform at both hierarchy levels. The result indicated that there was a main effect of hierarchy, *F* (1, 20) = 25.7, *p* < 0.001, η^2^ = 0.623. The CRN was greater at the lower-level of the hierarchy than the higher level (Fig. 5). There was a significant effect of caudality, *F* (1, 20) = 5.9, *p* < 0.05, η^2^ = 0.312. The CRN was greater at the front-central sites. There were neither other interactions, nor a main effect of laterality (all *p*s > 0.05).

## Discussion

In the present study, participants were required to maintain briefly a nested series of context cues in working memory in order to respond correctly to the targets or non-targets. Combining the two variables (stimulus type: target, non-target; hierarchy level: higher, lower), four conditions were formed: higher target, lower target, higher non-target, and lower non-target. The behavioral results indicated that participants correctly differentiated target sequences (e.g., 1-A-X) from non-targets (e.g., 1-A-Y), which was reflected in faster responses to target sequences than to non-target sequences. This behavioral result is consistent with Nee *et al*.^7^, which suggests that participants used higher- and lower-level context cues to form an expectation of the target sequence and the responses were slowed when this expectation was violated. That is, when the presented stimuli (e.g., Y) conflicted with their expectation, they would spend more time to make non-target response^7^,^13^. The electrophysiological results revealed a main effect of stimulus type, hierarchy level, and interactions between the two for N2 (220–350 ms) and P3 (350–500 ms) components.

### The higher the hierarchy level, the greater the uncertainty of the targets

For targets, the effect of hierarchy was observed for both the peak latency and peak amplitude of N2. The higher target elicited a delayed and increased N2 compared with the lower target. Previous studies suggested that uncertain cues produce larger N2 components than certain cues^40^,^41^,^42^,^43^. In the present study, the higher target (e.g., 1-***A***) could have been followed by the letter X or Y. If the letter X was shown, then the final stimulus (i.e., the circle) was a target; in contrast, if Y was shown then the final stimulus was a non-target. Therefore, for the higher targets, the decision and response preparation were uncertain. In contrast, the lower targets (e.g., 1-A-***X***) clearly specified what response should be given to the final stimulus. Thus, the difference in N2 amplitude might reflect the different certainty regarding the decision or behavior preparation between different hierarchical levels. That is, the current ERP results suggest that higher levels of a hierarchy are associated with greater uncertainty, resulting in an enhanced N2.

Alternatively, the uncertainty for the higher target might be interpreted as an underlying factor that evoked the more intensive process of conflict monitoring, because more competing responses are cued by the higher targets than the lower targets. The model of conflict monitoring proposed by Botvinick *et al*.^35^ suggests that an increased conflict monitoring process is associated with increased activation of the ACC, which is the source of the fronto-central N2^18^,^19^,^20^,^21^,^22^. The higher hierarchy the target is, the greater the uncertainty for the final response, resulting in increased conflict monitoring reflected by a larger N2 for the higher than the lower targets.

Consistent with previous imaging studies, the present study found that the hierarchical effect on the N2 component appeared in the frontal region, but was not found at the posterior scalp sites. This result supports the view that abstract representations occur primarily in frontal areas^8^,^9^,^12^,^44^.

After the N2 component, a clear P3 component was elicited by the higher and lower targets in the 350–500 ms time window. As expected, the lower targets elicited increased P3 amplitude as compared with higher targets. Previous studies have indicated that the P3 component reflects various fundamental cognitive processes^37^,^45^,^46^, and is linked to cognitive updating, memory encoding, retrieval, or decision making^36^,^37^,^38^,^47^,^48^,^49^. In the present study the P3 amplitudes differed significantly between the two hierarchies, which might reflect significant differences in context updating at different hierarchy levels. Specifically, at the lower hierarchy, the context cues were relatively more specific, and the cognitive update is more thorough. Conversely, at the higher hierarchy, the context cue was more abstract, and the cognitive update is less extensive. For a higher target (e.g., 1-***A***), there was uncertainty regarding whether the subsequent letter would be X or Y. Therefore, the appearance of the letter A could not lead to a clear prediction of the appearance of the final target; consequently, the cognitive context could not be comprehensively updated.

Recently, O’Connell and his colleagues analyzed the P300 component elicited by transient targets to examine its potential role as a “decision variable” signal that accumulates evidence toward a decision boundary. They demonstrated that centroparietal positivity signals a gradual, evidence-dependent approach to a fixed threshold level^48^,^49^. They suggested that the centroparietal positivity is equal to P300 and proposed that P300 can also be considered as a dynamically evolving neural signature of decision formation. This challenged the dominant explanatory accounts that conceived of P300 as a unitary neural event. In the present study, this decision-variable account can explain the difference in P3 amplitudes between higher and lower targets. That is, as compared with the higher targets, the lower targets appear at the end of the sequence and are the second match to the memory template. Consequently, the evidence confirming target identification is stronger at the lower hierarchical level relative to higher hierarchical level, which is reflected in an increased P3 amplitude for lower targets. Nevertheless, for non-targets, there is no difference in evidence accumulation toward decision making between higher non-targets and lower non-targets. That is, to make a non-target decision at a higher or lower hierarchical level, the only precondition is a mismatch between a stimulus (e.g., letter B or Y) and the memory template (e.g., 1-A-X). Accordingly, there should be no hierarchy effect for the P3 amplitudes elicited by non-targets. However, our results revealed a significant difference in P3 amplitudes between higher non-targets and lower non-targets. Therefore, the decision-variable account is unlikely to explain the results of the present study. Instead, the hierarchy effect on both targets and non-targets during the P3 component can be explained by an established proposal^37^,^47^,^50^ relating to stimulus evaluation and cognitive updating during P300. At the higher hierarchical level, the stimulus to be evaluated consisted of a number and a letter (e.g., 1-A or 1-B); in contrast, at the lower hierarchical level the stimulus to be evaluated consisted of a number and two letters (e.g., 1-A-X or 1-A-Y). The different amount information bound to the evaluated stimulus between different hierarchical levels might be an explanation for the hierarchical effect on P3 amplitude, which is in accordance with studies on memory manipulation^51^,^52^,^53^,^54^.

In brief, in a hierarchically structured task such as 1-A-X, the target response is more uncertain and the conflict monitoring of context cues of target response is more intensive at higher hierarchical levels, which is reflected in increased amplitude of N2 components. The stimulus information to be evaluated is greater and the context updating is more cognitively demanding at lower hierarchical levels, which is reflected in increased amplitude of P3 components.

### Conflict processing of non-targets at different hierarchy levels

Conflict monitoring, detection, and resolution are the main functions of the cognitive control system^35^,^55^,^56^,^57^, but the conflict processing of the targets might be different from that of non-targets. For the targets, the core of the conflict process is the online monitoring of the potential conflict. In contrast, for the non-targets, not only conflict monitoring but also conflict detection and resolution are required. The present study found that the hierarchy effect was reflected in responses to targets initiated during the N2 time window, with a larger N2 for the higher targets. Nevertheless, the hierarchy effect was not observed for the non-targets during the N2 time window. In order to exactly elucidate the hierarchy effect on the non-targets, we subtracted the potentials evoked by targets from that of non-targets. We named the peak of difference waveform CRN, because conflict processing was closely associated with the negative deflection evoked by non-targets. From the morphology, latency, amplitude, and scalp distribution, CRN is similar to FRN elicited primarily when there are conflicts, unexpected outcomes, and negative feedback^19^,^20^,^26^,^27^,^28^. In the present study, participants were required to differentiate targets from non-targets; as such, non-targets might have elicited stronger conflict and expectation violation than targets^7^,^13^. It is likely that the participants consciously maintained the two anticipated target sequences (e.g., 1-A-X, and 2-B-Y) in working memory, with all other sequences regarded as non-target sequences. Participants would not hold all the non-target sequences in working memory, because this would not be an optimal way to complete the task: It would be demanding of participants to remember additional non-target sequences. Accordingly, when the non-target appeared, they would be clearly inconsistent with the information held in working memory, and a larger conflict would be evoked, as reflected by a FRN-like negativity. This interpretation of the CRN is consistent with previous studies on the slot-machine task, in which participants experienced monetary gains and losses in the absence of responses^29^,^58^. In the gain condition of the slot-machine task, participants were told that they would gain some money every time three identical digits (xxx) were presented and that they would not gain anything whenever this was not the case (i.e., xyz or xxy). In the loss condition, participants were told that they would lose some money every time three identical digits (xxx) were presented to them and that they would not lose any money whenever this was not the case. The ERP results indicated that a FRN-like component was elicited whenever a stimulus was different from the preceding stimulus, reflecting the process of conflict detection^29^.

Importantly, the FRN-like negativity in the study of Donkers *et al*.^29^ was specifically larger if the third stimulus deviated from the preceding two stimuli (i.e., xxy trials) versus if the second stimulus deviated from the preceding stimulus (i.e., xyz trials), which might reflect the more intensive process of conflict detection of mismatching of stimuli to a consolidated memory template (i.e., xx). Similarly, the present study demonstrated that the amplitude of the CRN differed markedly between different hierarchy levels. The peak amplitude of CRN for the lower-level hierarchy was approximately four times that of the higher-level hierarchy. Two factors contributed to larger CRN-amplitude at lower hierarchy levels than at higher hierarchy levels. One factor is the larger difference in the N2 peak amplitude, and the other is the delayed P300. For the N2 peak amplitude, the difference between non-target and target is about 1.8 μV at the lower hierarchy level and about 0.7 μV at the higher hierarchy level. Because of the longer P300 latency for the non-target, the CRN-amplitude increased dramatically after the N2 and reached the maximal point at about 320 ms in the lower hierarchy level. At the higher hierarchy level, the P300 latency was also longer for the non-target than the target, and the CRN-amplitude also increased after the N2, but the maximum size of the CRN-amplitude was significantly smaller at the higher hierarchy level than at the lower hierarchy level. The larger CRN at the lower level of the hierarchy indicates that the conflict detection and the subsequent conflict resolution might be much more intensive at the lower level of the hierarchy than at the higher level of the hierarchy. For example, after seeing the digit 1, participants might have immediately retrieved the memory template (1-A-X) and expected the next letter would be an A. A letter B would conflict with the memory template and would conflict with participants’ expectations. For the lower-level hierarchy, participants maintained the number 1 and letter A in working memory. They would expect the next letter would be an X. At this point, the participant had expected the final target for a longer time than they did at the higher hierarchy level, and the end of the target sequence was close, so that the target expectation at the lower hierarchical level might be greater than at the higher hierarchical level. Moreover, the memory template would have been consolidated after the first match (i.e., matching the letter A to 1-A-X), so a mismatched stimulus (e.g., Y) might elicit a more intensive conflict with the consolidated memory at the lower hierarchical level. Taken together, the cognitive control system regarded the non-target as a clear conflict with the consolidated memory template, resulting in an enhanced FRN-like negativity^23^,^59^. The conflict processing is more intensive at the lower and concrete nodes.

It is necessary to acknowledge that participants in the present study performed a variant of 1-2-AX-CPT task^7^,^13^,^60^, which has previously been used to show hierarchical effects. However, while the sequences can be described in a hierarchical fashion, it remains unclear whether participants represent the different context cues in a hierarchical way. Alternatively, they might have simply represented the stimuli as eight separate sequences, or three kinds of sequence including two target sequences and non-target sequences. Future studies are needed to support the hierarchical representation of the 1-2-A task by providing additional empirical evidence.

## Conclusion

In the present study, we used a hierarchically structured task and examined the ERPs evoked by targets and non-targets at different hierarchical levels. Results showed that the two ERP components, N2 and P3, were modulated by the hierarchical level and stimulus type. A greater N2 and smaller P3 were induced by the higher- as compared with the lower-level of the hierarchy. Moreover, a larger CRN was induced by the lower level of the hierarchy as compared with the higher level, reflecting a more intensive conflict processing. These results provide new evidence regarding the hierarchical representations within the human brain.

## Methods

### Participants

Twenty-one paid volunteers (thirteen females) aged 20–27 years (mean 23.7 years) participated in this study. All participants were right-handed, had normal or corrected-to-normal vision, and did not report any history of psychiatric or neurological illness. Participants provided written informed consent before the experiment. The study was approved by the Ethics Committee of Liaoning Normal University (China), and the investigation was carried out in accordance with the latest version of the Declaration of Helsinki.

### Materials and design

**Figure 1:**
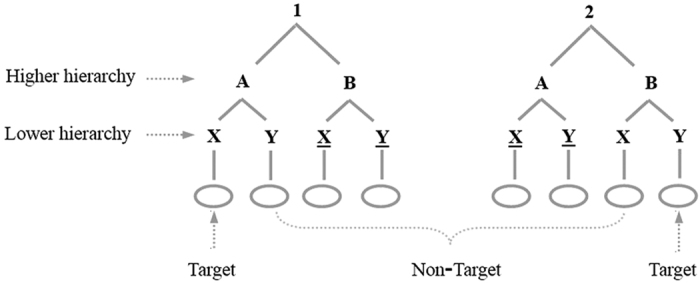
Illustration of the task hierarchy. Participants responded to circles based upon a nested series of context cues. The data for the underlined letters were not analyzed since participants had identified the sequence as non-targets before the appearance of these letters.

The experimental task was modified from Nee and Brown^13^, and constructed based upon a nested series of context cues (Fig. 1). Under the number 1 context, participants should make a target response to the response stimulus (i.e., an empty circle) if 1 was followed by the letters A and X, and make a non-target response otherwise. Under the number 2 context, participants should make a target response to the response stimulus if 2 was followed by letters B and Y, and make a non-target response otherwise. Hence, in order to respond correctly, participants had to maintain the hierarchy context (e.g., 1-A-X) in working memory.

The distance between the participant’s eyes and the screen was approximately 1.2 m. The horizontal and vertical dimensions of each stimulus were less than 3.5° of visual angle.

The experiment consisted of a practice and a formal experiment session. The practice consisted of 20 trials to familiarize the participants with the experimental procedure. The formal experiment consisted of 8 blocks, each of 40 trials. Each of the eight sequences (1-A-X, 1-A-Y, 1-B-X, 1-B-Y, 2-A-X, 2-A-Y, 2-B-X, and 2-B-Y) was presented for 40 trials, which were randomly assigned among blocks. The formal experiment lasted just under 30 min. Participants were permitted to take a rest after finishing one block. All stimuli were presented on a computer screen with a resolution of 800 × 600 pixels, against a silver-gray background. Participants were required to judge whether the sequentially presented stimuli were the two target-directed sequences (i.e., 1-A-X or 2-B-Y) or non-target directed sequences, and they should make a response to the response stimuli (e.g., an empty circle), by pressing one of two keys (e.g. press F to targets and J to non-targets).The assignment of response keys to target and non-target was balanced across subjects. Participants were instructed to respond rapidly and accurately.

### Procedure

As shown in Fig. 6, the beginning of the trial was indicated by a “+” sign in the center of screen for 500 ms, followed by a blank screen for 800–1200 ms. Then, three context stimuli (i.e., one number and two letters) were displayed sequentially. Each stimulus was displayed for 100 ms with an 800–1200 ms interstimulus interval. The first stimulus was a number 1 or 2, the second the letter A or B, and the third the letter X or Y. Finally, a circle was displayed for up to 2000 ms or disappeared when the participant responded. The experiment was implemented using E-Prime software (Version 2.0; Psychological Software Tools, Pittsburgh, PA).

### Electrophysiological recording and analysis

Electrophysiological activity was recorded using a 64-channel EEG system (Brain Products GmbH, Munich, Germany) with TP9 and TP10 as reference electrodes. A ground electrode (AFz) was placed on the medial aspect of the frontal region, between Fz and Fpz, and between AF3 and AF4. The vertical electrooculogram (VEOG) was recorded supra- and infra-orbitally at the left eye, and the horizontal electrooculogram (HEOG) was recorded as the voltage between electrodes placed 1 cm to the left and right of the external canthi. Ocular artifacts were monitored by electrodes placed below the left eye (VEOG) and on the right side of the right eye (HEOG). The resistance of the electrodes was kept below 10 kΩ. The EEG and EOG were amplified using a 0.01–100 Hz bandpass and were continuously sampled at 500 Hz/channel. Trials with EOG artifacts (mean EOG voltage exceeding ±80 μV) and those contaminated with artifacts due to amplifier clipping, bursts of electromyographic (EMG) activity, or peak-to-peak deflection exceeding ±80 μV were excluded from averaging.

**Figure 2:**
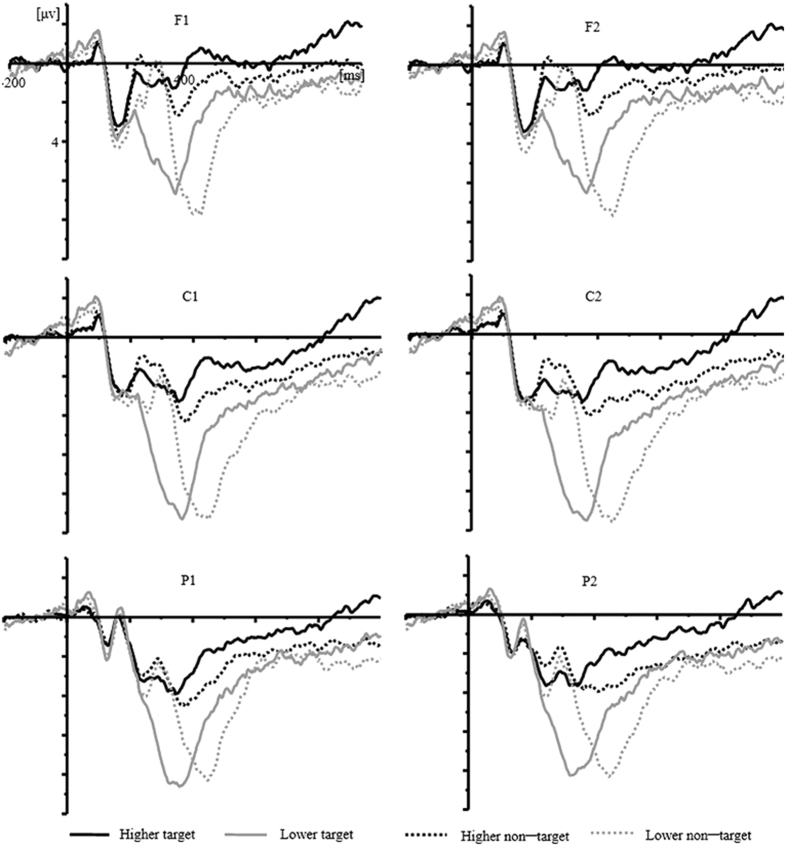
Grand average (n = 21) ERP waveforms for four conditions (higher-level target, lower-level target, higher-level non-target, lower-level non-target) at the selected electrode sites.

**Figure 3:**
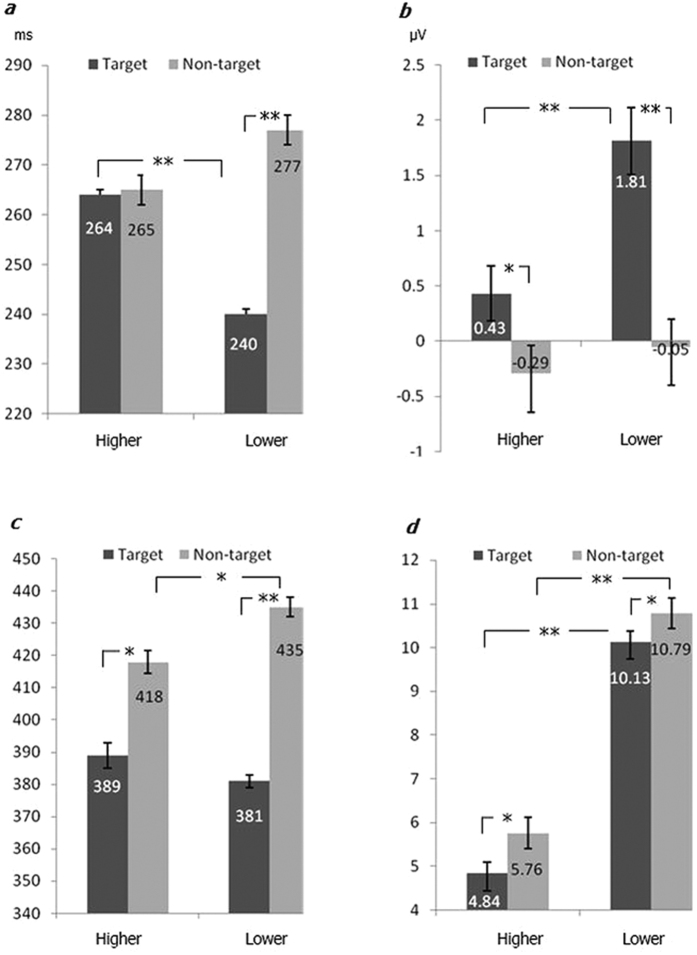
The peak latencies and amplitudes (across all 24 electrodes) of N2 and P3 under different conditions. (**a**) results of N2 latency; (**b**) results of N2 amplitude; (**c**) results of P3 latency; (**d**) results of P3 amplitude. *denotes *p* < 0.05; **denotes *p* < 0.01.

**Figure 4:**
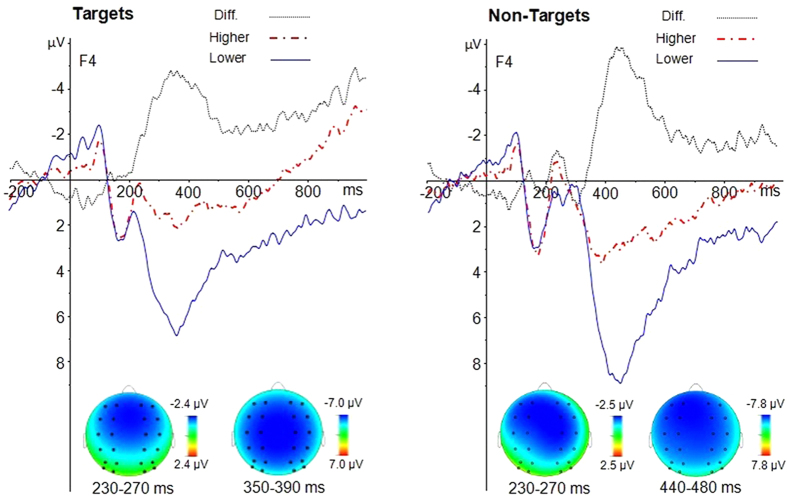
Hierarchy effect on targets and non-targets. Left panel: Difference waves (higher-level minus lower-level) and topographies for targets. Right panel: Difference waves (higher-level minus lower-level) and topographies for non-targets.

**Figure 5:**
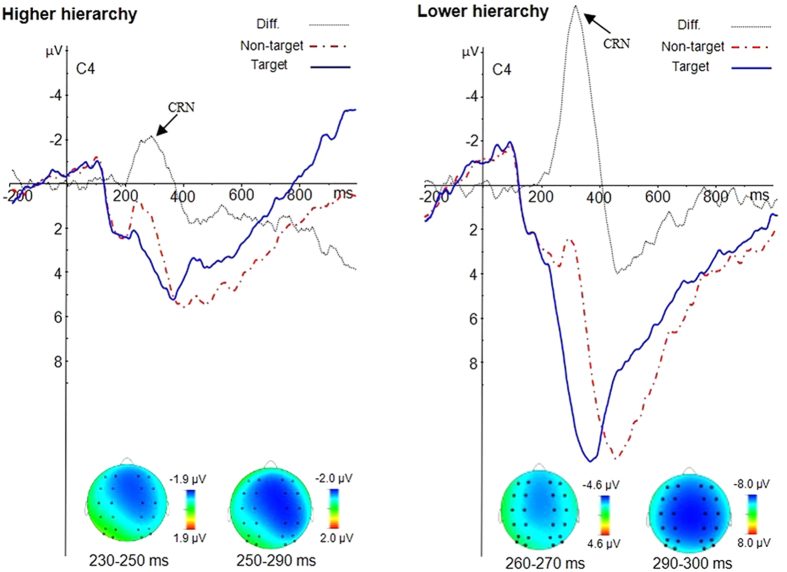
Type effect at higher- and lower-level hierarchies. Left panel: Difference waves (non-target minus target) and topographies at higher-level hierarchies. Right panel: Difference waves (non-target minus target) and topographies at lower-level hierarchies.

**Figure 6:**
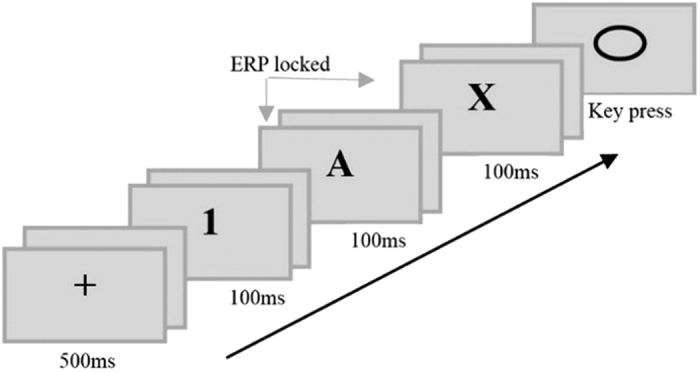
Experimental procedure for one trial. Each blank interval lasted 800–1200 ms.

Data were collected continuously and analyzed off-line using the Brain Vision Analyzer software (Brain Products, Munich, Germany). Frequencies lower than 0.1 Hz or higher than 30 Hz were digitally filtered (24 dB) from the ERPs. The analysis epoch for the ERP was 1200 ms, time-locked to the letter stimuli, including a 200 ms pre-stimulus baseline. Only those segments with a correct judgment were averaged. On the basis of the grand averaged ERPs and topographical map (Figs 2,4 and 5), the following 24 electrode sites in three regions were chosen for statistical analysis: the frontal region, consisting of F1, F3, FC1, FC3, F2, F4, FC2, and FC4; the central region, consisting of C1, C3, CP1, CP3, C2, C4, CP2, and CP4; and the posterior region, consisting of P1, P3, PO3, PO7, P2, P4, PO4, and PO8. The peak amplitudes and latencies of N2 (220–350 ms) and P3 (350−500 ms) were analyzed using a 2 (hierarchy: higher level vs. lower level) ×2 (type: target vs. non-target) ×2 (laterality: left vs. right) ×3 (caudality: frontal, central, posterior) ×4 (electrode) analysis of variance (ANOVA). To isolate the difference between target and non-target trials, we followed the same method as Miltner *et al*.^39^. We subtracted the target waveform from the non-target waveform, and this subtraction procedure removed contributions of any components that were elicited in response to both types of stimuli, leaving only those components that were specifically related to CRN. Then, we examined specifically the hierarchical effect on the CRN by conducting a 2 (hierarchy: higher vs. lower level) ×2 (laterality: left vs. right) ×3 (caudality: frontal, central, posterior) ×4 (electrode) ANOVA on the peak amplitudes of CRN. For all analyses, *p*-values were corrected according to the Greenhouse-Geisser method.

## Additional Information

**How to cite this article**: Cao, B. *et al*. Hierarchical effects on target detection and conflict monitoring. *Sci. Rep*. **6**, 32234; doi: 10.1038/srep32234 (2016).

## References

[b1] Kundey S. M. A. & Rowan J.D. Hierarchical organization in serial pattern learning. Learn Motiv. 46, 60–68 (2014).

[b2] Kundey S. M. A. . Involvement of working memory in college students’ sequential pattern learning and performance. Learn Motiv. 44, 114–126 (2013).

[b3] Ranti C., Chatham C. H. & Badre D. Parallel temporal dynamics in hierarchical cognitive control. Cognition 142, 205–229 (2015). 26051820 10.1016/j.cognition.2015.05.003 PMC4500760

[b4] Schneider D. W. & Logan G. D. Hierarchical control of cognitive processes: Switching tasks in sequences. J. Exper Psychol. G. 135, 623–640 (2006). 10.1037/0096-3445.135.4.623 17087577

[b5] Weaver S. M. & Arrington C. M. The effect of hierarchical task representations on task selection in voluntary task switching. J. Exper Psychol. 39, 1128–1141 (2013). 10.1037/a0031677 23421506

[b6] Zarr N. & Brown J. W. Hierarchical error representation in medial prefrontal cortex. NeuroImage 124, 238–247 (2016). 26343320 10.1016/j.neuroimage.2015.08.063

[b7] Nee D. E., Jahn A. & Brown J. W. Prefrontal cortex organization: dissociating effects of temporal abstraction, relational abstraction, and integration with FMRI. Cerebral Cortex 24(9), 2377–2387 (2014). 23563962 10.1093/cercor/bht091 PMC4184367

[b8] Badre D. Cognitive control, hierarchy, and the rostro-caudal organization of the frontal lobes. Trends in Cogn Sci. 12, 193–200 (2008). 18403252 10.1016/j.tics.2008.02.004

[b9] Badre D. & D’Esposito M. Functional magnetic resonance imaging evidence for a hierarchical organization of the prefrontal cortex. J. Cogn Neurosci. 19, 2082–2099 (2007). 17892391 10.1162/jocn.2007.19.12.2082

[b10] Bahlmann J., Blumenfeld R. S. & D’Esposito M. The rostro-caudal axis of frontal cortex is sensitive to the domain of stimulus information. Cerebral Cortex 25, 7 (2014). 10.1093/cercor/bht419 PMC4459285 24451658

[b11] Diuk C., Tsai K., Wallis J., Botvinick M. & Niv Y. Hierarchical learning induces two simultaneous, but separable, prediction errors in human basal ganglia. J. Neurosci. 33, 5797–5805 (2013). 23536092 10.1523/JNEUROSCI.5445-12.2013 PMC3865543

[b12] Koechlin E., Ody C. & Kouneiher F. The architecture of cognitive control in the human prefrontal cortex. Science 302, 1181–1185 (2003). 14615530 10.1126/science.1088545

[b13] Nee D. E. & Brown J. W. Rostral-caudal gradients of abstraction revealed by multi-variate pattern analysis of working memory. NeuroImage 63, 1285–1294 (2012). 22992491 10.1016/j.neuroimage.2012.08.034 PMC3472084

[b14] Unger K. & Badre D. Hierarchical reinforcement learning. Brain Map. 41, 367–373 (2015).

[b15] Brasted P. J. & Wise S. P. Comparison of learning-related neuronal activity in the dorsal premotor cortex and striatum. Eur. J. Neurosci. 19, 721–740 (2004). 14984423 10.1111/j.0953-816x.2003.03181.x

[b16] Hoshi E. & Tanji J. Differential involvement of neurons in the dorsal and ventral premotor cortex during processing of visual signals for action planning. J. Neurophysiol. 95, 3596–3616 (2006). 16495361 10.1152/jn.01126.2005

[b17] Lucchetti C. & Bon L. Time-modulated neuronal activity in the premotor cortex of macaque monkeys. Exper Brain Res. 141, 254–260 (2001). 11713637 10.1007/s002210100818

[b18] Gajewski P. D., Stoerig P. & Falkenstein M. ERP Correlates of response selection in a response conflict paradigm. Brain Res. 1189, 127–134 (2008). 18053974 10.1016/j.brainres.2007.10.076

[b19] Heldmann M., Rüsseler J. & Münte T. F. Internal and external information in error processing. BMC Neurosci. 9, 1–8 (2008). 18366727 10.1186/1471-2202-9-33 PMC2291472

[b20] Mushtaq F., Wilkie R. M., Mon-Williams M. A. & Schaefer A. Randomised prior feedback modulates neural signals of outcome monitoring. NeuroImage 125, 868–879 (2016). 26497268 10.1016/j.neuroimage.2015.10.046 PMC4692517

[b21] Pfabigan D. M. . Context-sensitivity of the feedback-related negativity for zero-value feedback outcomes. Biol Psychol. 104, 184–192 (2015). 25541513 10.1016/j.biopsycho.2014.12.007

[b22] Van,Veen V. & Carter C. S. The timing of action-monitoring processes in the anterior cingulate cortex. J. Cogn Neurosci. 14, 593–602 (2002). 12126500 10.1162/08989290260045837

[b23] Lange J. J., Wijers A. A., Mulder L. J. M. & Mulder G. Color selection and location selection in ERPs: Differences, similarities and ‘neural specificity’. Biol Psychol. 48, 153–182 (1998). 9700016 10.1016/s0301-0511(98)00011-8

[b24] Azizian A., Freitas A. L., Parvaz M. A. & Squires N. K. Beware misleading cues: Perceptual similarity modulates the N2/P3 complex. Psychophysiology 43, 253–260 (2006). 16805863 10.1111/j.1469-8986.2006.00409.x

[b25] Pfefferbaum A., Ford J. M., Weller B. J. & Kopell B. S. ERPs to response production and inhibition. Electroencephal Clini Neurophysiol. 60, 423–434 (1985). 10.1016/0013-4694(85)91017-x 2580694

[b26] Hauser T. U. . The feedback-related negativity (FRN) revisited: New insights into the localization, meaning and network organization. NeuroImage 84, 159–168 (2014). 23973408 10.1016/j.neuroimage.2013.08.028

[b27] Holroyd C. B. & Coles M. G. H. The neural basis of human error processing: Reinforcement learning, dopamine, and the error-related negativity. Psychol Rev. 109, 679–709 (2002). 12374324 10.1037/0033-295X.109.4.679

[b28] Nieuwenhuis S., Holroyd C. B., Mol N. & Coles M. G. H. Reinforcement-related brain potentials from medial frontal cortex: Origins and functional significance. Neurosci Biobeh Rev. 28, 441–448 (2004). 10.1016/j.neubiorev.2004.05.003 15289008

[b29] Donkers F. C. L., Nieuwenhuis S. & van Boxel G. J. M. Mediofrontal negativities in the absence of responding. Cogn Brain Res. 25, 777–787 (2005). 10.1016/j.cogbrainres.2005.09.007 16249075

[b30] Kotchoubey B. I., Jordan J. S., Grozinger B., Westphal K. P. & Kornhuber H. H. Event-related brain potentials in a varied set memory search task: A reconsideration. Psychophysiology 33, 530–540 (1996). 8854740 10.1111/j.1469-8986.1996.tb02429.x

[b31] Kramer A. F., Strayer D. L. & Buckley J. Task versus component consistency in the development of automatic processing: A psychophysiological assessment. Psychophysiology 28, 425–437 (1991). 1745722 10.1111/j.1469-8986.1991.tb00726.x

[b32] Wang Y., Cui L., Wang H., Tian S. & Zhang X. The sequential processing of visual feature conjunction mismatches in the human brain. Psychophysiology 41, 21–29 (2004). 14692997 10.1111/j.1469-8986.2003.00134.x

[b33] Wang Y. . Event-related potentials evoked by multi-feature conflict under different attentive conditions. Exper Brain Res. 148, 451–457 (2003). 12582828 10.1007/s00221-002-1319-y

[b34] Folstein J. R. & Petten C. V. Influence of cognitive control and mismatch on the N2 component of the ERP: A review. Psychophysiology 45(1), 152–170 (2008). 17850238 10.1111/j.1469-8986.2007.00602.x PMC2365910

[b35] Botvinick M. M., Braver T. S., Barch D. M., Carter C. S. & Cohen J. D. Conflict monitoring and cognitive control. Psychological review 108(3), 624–652 (2001). 11488380 10.1037/0033-295x.108.3.624

[b36] Duncan-Johnson C. C. & Donchin E. The P300 component of the event–related brain potential as an index of information processing. Biol Psychol. 14, 1–52 (1982). 6809064 10.1016/0301-0511(82)90016-3

[b37] Donchin E. & Coles M. G. H. Is the P300 component a manifestation of context updating? Behav Brain Sci. 11, 357–374 (1988).

[b38] Ferdinand N. K., Mecklinger A. & Opitz B. Learning context modulates the processing of expectancy violations. Brain Res. 1629, 72–84 (2015). 26475976 10.1016/j.brainres.2015.10.017

[b39] Miltner W. H. R., Braun C. H. & Coles M. G. H. Event-related brain potentials following incorrect feedback in a time-estimation task: evidence for a generic neural system for error detection. J. Cogn Neurosci. 9, 788–798 (1997). 23964600 10.1162/jocn.1997.9.6.788

[b40] Bland A. R. & Schaefer A. Electrophysiological correlates of decision making under varying levels of uncertainty. Brain Res. 1417, 55–66 (2011). 21911213 10.1016/j.brainres.2011.08.031

[b41] Lin H. Y. . Larger N2 and smaller early contingent negative variation during the processing of uncertainty about future emotional events. Int. J. Psychophysiol. 94, 292–297 (2014). 25312204 10.1016/j.ijpsycho.2014.10.004

[b42] Polezzi D., Sartori G., Rumiati R., Vidotto G. & Daum I. Brain correlates of risky decision-making. NeuroImage 49, 1886–1894 (2010). 19761850 10.1016/j.neuroimage.2009.08.068

[b43] Xu Q. . How an uncertain cue modulates subsequent monetary outcome evaluation: an ERP study. Neurosci Lett. 505(2), 200–204 (2011). 22027182 10.1016/j.neulet.2011.10.024

[b44] Badre D., Hoffman J., Cooney J. W. & D’Esposito M. Hierarchical cognitive control deficits following damage to the human frontal lobe. Nat Neurosci. 12, 515–522 (2009). 19252496 10.1038/nn.2277 PMC2990342

[b45] Picton T. W. The P300 wave of the human event-related potential. J. ClinNeurophysiol. 9, 456–479 (1992). 10.1097/00004691-199210000-00002 1464675

[b46] Polich J. Cognitive brain potentials. Curr Dir Psychol Sci. 2, 175–179 (1993).

[b47] Kutas M., McCarthy G. & Donchin E. Augmenting mental chronometry: The P300 as a measure of stimulus evaluation time. Science 197, 792–795 (1977). 887923 10.1126/science.887923

[b48] O’Connell R. G., Dockree P. M. & Kelly S. P. A supramodal accumulation-to-bound signal that determines perceptual decisions in humans. Nat Neurosci. 15, 1729–1737 (2012). 23103963 10.1038/nn.3248

[b49] Kelly S. P. & O’Connell R. G. Internal and External Influences on the Rate of Sensory Evidence Accumulation in the Human Brain. J. Neurosci. 33(50), 19434–19441 (2013). 24336710 10.1523/JNEUROSCI.3355-13.2013 PMC6618757

[b50] Duncan-Johnson C. C. & Donchin E. On quantifying surprise: The variation of event-related potentials with subjective probability. Psychophysiology 14, 456–467 (1977). 905483 10.1111/j.1469-8986.1977.tb01312.x

[b51] Gao H. M. . Two stages of directed forgetting: Electrophysiological evidence from a short-term memory task. Psychophysiology 53(6), 806–813 (2016). 26892593 10.1111/psyp.12628

[b52] Kok A. On the utility of P3 amplitude as a measure of processing capacity. Psychophysiology 38(3), 557–577 (2001). 11352145 10.1017/s0048577201990559

[b53] Kusak G., Grune K., Hagendorf H. & Metz A. M. Updating of working memory in a running memory task: an event-related potential study. Int. J. Psychophysiol. 39, 51–65 (2000). 11120347 10.1016/s0167-8760(00)00116-1

[b54] Shucard J. L., Tekok-Kilic A., Shiels K. & Shucard D. W. Stage and load effects on ERP topography during verbal and spatial working memory. Brain res. 1254, 49–62 (2009). 19083994 10.1016/j.brainres.2008.11.063 PMC2909776

[b55] Botvinick M. M., Cohen J. D. & Carter C. S. Conflict monitoring and anterior cingulate cortex: An update. Trends in Cogn Sci 8, 539–546 (2004). 15556023 10.1016/j.tics.2004.10.003

[b56] Egner T. & Hirsch J. Cognitive control mechanisms resolve conflict through cortical amplification of task-relevant information. Nat Neurosci. 8, 1784–1790 (2005). 16286928 10.1038/nn1594

[b57] Walsh B. J., Buonocore M. H., Carter C. S. & Mangun G. R. Integrating conflict detection and attentional control mechanisms. J. Cogn Neurosci. 23, 2211–2221 (2011). 21126158 10.1162/jocn.2010.21595 PMC3142580

[b58] Donkers F. C. L. & van Boxtel G. J. M. Mediofrontal negativities to averted gains and losses in the slot-machine task: a further investigation, J. Psychophysiol. 19, 256–262 (2005).

[b59] Bellebaum C., Polezzi D. & Daum I. It is less than you expected: The feedback-related negativity reflects violations of reward magnitude expectations. Neuropsychologia 48(11), 3343–3350 (2010). 20655319 10.1016/j.neuropsychologia.2010.07.023

[b60] Frank M. J., Loughry B. & O’Reilly R. C. Interactions between frontal cortex and basal ganglia in working memory: a computational model. Cogn Affect Behav Neurosci. 1, 137–160 (2001). 12467110 10.3758/cabn.1.2.137

